# Kartogenin prevents cartilage degradation and alleviates osteoarthritis progression in mice via the miR-146a/NRF2 axis

**DOI:** 10.1038/s41419-021-03765-x

**Published:** 2021-05-13

**Authors:** Mingzhuang Hou, Yijian Zhang, Xinfeng Zhou, Tao Liu, Huilin Yang, Xi Chen, Fan He, Xuesong Zhu

**Affiliations:** 1 Department of Orthopaedics, The First Affiliated Hospital of Soochow University, Soochow University, Suzhou, China; 2 Orthopaedic Institute, Medical College, Soochow University, Suzhou, China; 3 Department of Pathology, The Third Affiliated Hospital of Soochow University, Changzhou, China

**Keywords:** Osteoarthritis, Drug development

## Abstract

Osteoarthritis (OA) is a common articular degenerative disease characterized by loss of cartilage matrix and subchondral bone sclerosis. Kartogenin (KGN) has been reported to improve chondrogenic differentiation of mesenchymal stem cells. However, the therapeutic effect of KGN on OA-induced cartilage degeneration was still unclear. This study aimed to explore the protective effects and underlying mechanisms of KGN on articular cartilage degradation using mice with post-traumatic OA. To mimic the in vivo arthritic environment, in vitro cultured chondrocytes were exposed to interleukin-1β (IL-1β). We found that KGN barely affected the cell proliferation of chondrocytes; however, KGN significantly enhanced the synthesis of cartilage matrix components such as type II collagen and aggrecan in a dose-dependent manner. Meanwhile, KGN markedly suppressed the expression of matrix degradation enzymes such as MMP13 and ADAMTS5. In vivo experiments showed that intra-articular administration of KGN ameliorated cartilage degeneration and inhibited subchondral bone sclerosis in an experimental OA mouse model. Molecular biology experiments revealed that KGN modulated intracellular reactive oxygen species in IL-1β-stimulated chondrocytes by up-regulating nuclear factor erythroid 2-related factor 2 (NRF2), while barely affecting its mRNA expression. Microarray analysis further revealed that IL-1β significantly up-regulated miR-146a that played a critical role in regulating the protein levels of NRF2. KGN treatment showed a strong inhibitory effect on the expression of miR-146a in IL-1β-stimulated chondrocytes. Over-expression of miR-146a abolished the anti-arthritic effects of KGN not only by down-regulating the protein levels of NRF2 but also by up-regulating the expression of matrix degradation enzymes. Our findings demonstrate, for the first time, that KGN exerts anti-arthritic effects via activation of the miR-146a-NRF2 axis and KGN is a promising heterocyclic molecule to prevent OA-induced cartilage degeneration.

## Introduction

Osteoarthritis (OA) is one of the most common degenerative joint disorders in elderly people. Mild or moderate OA is characterized by pain and stiffness of the joints but severe OA can cause patients disabled^1^. Current treatments using nonsteroidal anti-inflammatory drugs such as NSAIDs can only alleviate associated symptoms but fail to prevent OA progression. Degradation of articular cartilage as the hallmark of OA pathogenesis can be caused by excessive loss of cartilage extracellular matrix (ECM). Type II collagen (COL II) and aggrecan are two major components of cartilage ECM. COL II regulates the metabolic balance whereas aggrecan confers resistance to tension or compression at the cartilage region^2^. However, patients with OA have dysfunctional chondrocytes that release excessive degrading enzymes including matrix metalloproteinase 13 (MMP13) and a disintegrin metalloproteinase with thrombospondin motifs 5 (ADAMTS5), which further aggravate the damage of articular cartilage^3^. This underlines the urgent needed to develop new and effective interventions against cartilage degeneration.

Kartogenin (KGN) is a small bioactive molecule that was first reported to promote chondrogenic differentiation of bone marrow mesenchymal stem cells (MSCs)^4^. By breaking the core-binding factor β (CBFβ) and filamin A, KGN promotes the nuclear translocation of CBFβ to form a complex with runt-related transcription factor-1 (RUNX1) and enhances the expression of COL II and aggrecan. During limb development, KGN improves the development of cartilage nodule and synovial joint through the transforming growth factor β (TGFβ) signaling pathway^5^. To investigate the potential of KGN in promoting cartilage regeneration, Shi et al. encapsulated KGN within ultraviolet-cross-linkable hydrogel nanoparticles and demonstrated that the KGN-hydrogel complex enhanced cartilage matrix formation in rabbits^6^. In addition, KGN has been shown beneficial effects on the formation of meniscus-like tissue in vitro using tendon MSCs and regeneration of tendon graft in vivo^7^. In our recent study, we found KGN repressed production of reactive oxygen species (ROS) and improved cellular antioxidant functions in human bone marrow MSCs by up-regulating the expression of silent information regulator type 1 (SIRT1)^8^, suggesting that KGN may ameliorate OA-induced cartilage degradation by attenuating oxidative stress.

Emerging evidence indicates that excessive production of ROS can impair cartilage redox homeostasis and thus results in cartilage matrix degradation. During OA pathogenesis, the high levels of superoxide anion radical (O_2_^-^), hydroxyl radical (HO^-^) and hydrogen peroxide (H_2_O_2_) can not only inhibit the synthesis of cartilage matrix proteins but also increase the expression of several matrix degradation enzymes^9^. Nuclear factor erythroid 2-related factor 2 (NRF2), as a key transcription regulator of antioxidant defense system, plays a critical role in regulating more than 200 cytoprotective genes. In response to oxidative stimulus, NRF2 can be translocated into the nucleus, bind to antioxidant response element (ARE), and activate the transcription of antioxidant enzymes such as such as superoxide dismutase 1 (SOD1) and 2, catalase, heme oxygenase-1 (HO-1) and glutathione peroxidase 1 (GPX1)^10^. Over-expression of NRF2 has been shown to inhibit apoptosis and mitochondrial dysfunction caused by interleukin-1β (IL-1β) in human OA chondrocytes. Activation of the NRF2/HO-1 axis successfully prevents cartilage destruction in mice with surgically induced OA by inhibiting the formation of Nod-like receptor pyrin domain 3 (NLRP3) inflammasomes and lipopolysaccharide (LPS)-induced chondrocyte pyroptosis^11^. In contrast, knockdown of NRF2 leads to over-production of ROS in chondrocytes and results in cell death^12^. More importantly, impairment of the NRF2-related antioxidant system enhances inflammatory responses in patients with type 2 diabetes and aggravates the OA progression^13^. However, it is still unknown whether KGN could prevent OA-induced cartilage degeneration via the NRF2-mediated antioxidant signaling pathways.

MicroRNAs (miRNAs) are small (18-25 nucleotide) noncoding RNAs that regulate numerous biological processes via post-transcriptional modulation of gene expression. Several microRNAs have been identified to regulate cartilage degeneration in patients with OA. For instance, Nakamura et al. demonstrated that miR-181a-5p mediated the destruction of knee OA cartilage. Accordingly, treatment with miR-181a-5p antisense oligonucleotides attenuated cartilage destruction by down-regulating the expression of apoptotic, catabolic, and hypertrophic markers^14^. In addition, Endisha et al. revealed that the expression of miR-34a-5p was significantly increased in the late stages of OA. Animal experiments reveal that intra-articular injection with miR-34a-5p antisense-oligonucleotide cushions against cartilage destruction, suggesting the therapeutic potential of miR-34a-5p^15^. In our previous study, we found melatonin exerts its anti-arthritic effect by suppressing the expression of MMP13 and ADAMTS5 via targeting miR-140-5p^16^.

Several microRNAs have been reported to regulate the translation of NRF2 mRNA. Among them, microRNA-146a (miR-146a) has been shown to suppress NRF2 expression by binding to the 3′-untranslated regions (3′-UTRs) of NRF2 mRNA. It has been reported that, in the livers of aged rats, high levels of miR-146a inhibit secretion of NRF2 to disrupt the down-stream detoxification system^17^. Cheleschi et al demonstrated that, in human OA chondrocytes, hydrogen peroxide-induced oxidative stress up-regulated the expression of NRF2 and the related antioxidant enzymes via miR-146a^18^. On the other hand, knockout or inhibition of miR-146a has been demonstrated to ameliorate articular cartilage destruction in an experimental OA mouse model^19^. However, the effects of KGN on OA-associated microRNAs and the underlying mechanisms remain to be elucidated.

In this study, we hypothesized that KGN could inhibit OA-induced degeneration of articular cartilage by enhancing the antioxidant functions of chondrocytes. In vitro OA microenvironment was induced by exposing human chondrocytes to IL-1β. To evaluate the protective effects of KGN on cartilage degeneration in vivo, mice with posttraumatic OA were injected intra-articularly with KGN. The underlying mechanisms involving NRF2 and miR-146a signaling pathways were also investigated.

## Results

### KGN suppressed the expression of matrix degradation enzymes in human chondrocytes

**Fig. 1:**
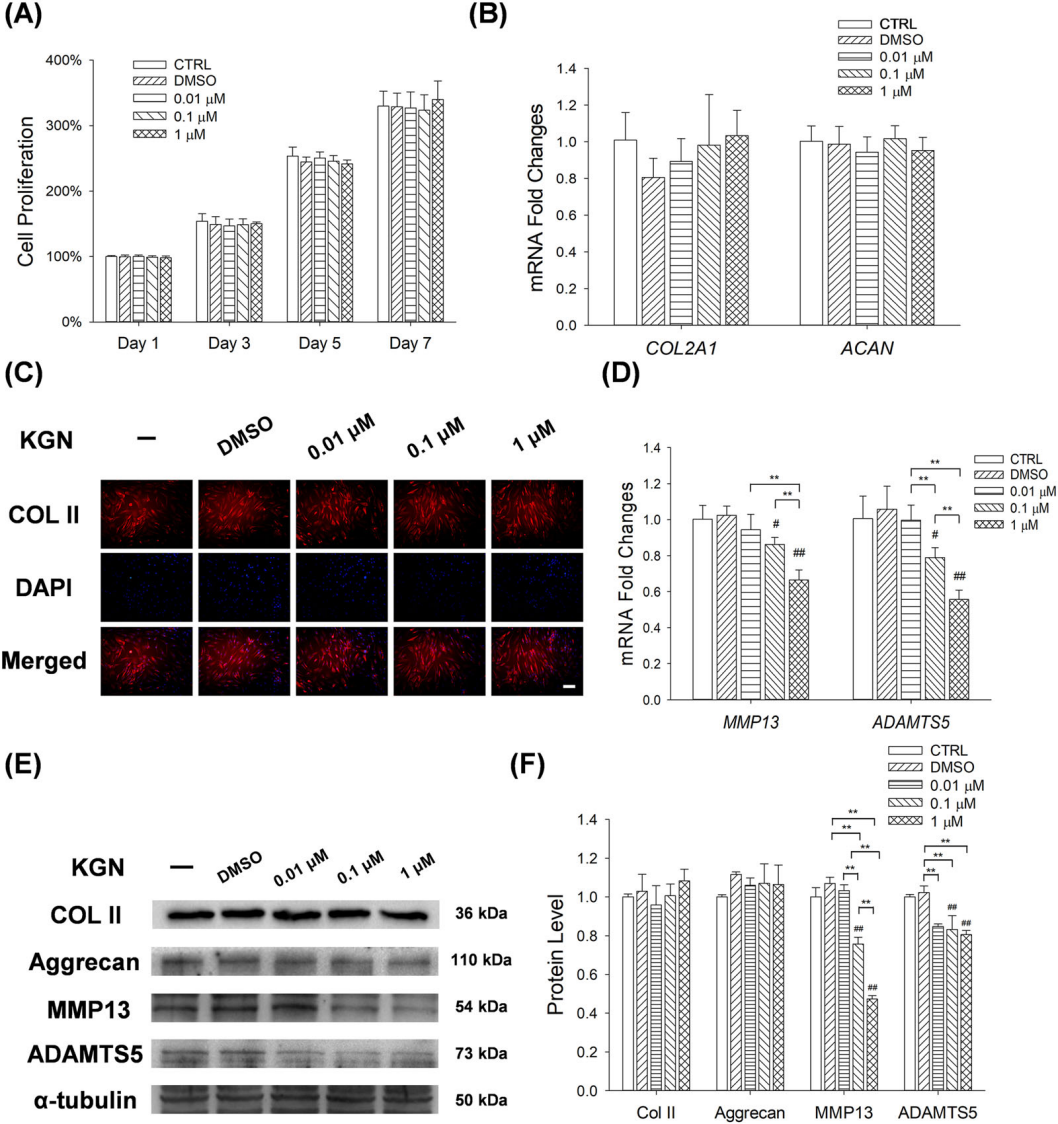
The effects of KGN on proliferation as well as anabolic and catabolic metabolism of human articular chondrocytes. **A** Proliferation of chondrocytes after 1, 3, 5 and 7 days of KGN treatment. **B** RT-PCR analysis for the expression of *COL2A1* and *ACAN* mRNA levels in chondrocytes after KGN treatment. **C** Immunofluorescence analysis for the expression of COL II proteins in KGN-treated chondrocytes. Scale bar = 100 μm. **D** RT-PCR analysis for the expression of *MMP13* and *ADAMTS5* mRNA levels in chondrocytes after KGN treatment. **E–F** The effects of KGN on the protein levels of COL II, aggrecan, MMP13 and ADAMTS5 in normal chondrocytes were determined using Western blot assays. Cells were treated with 0.01 μM, 0.1 μM or 1 μM of KGN. Values represent mean ± S.E.M of six replicas for cell proliferation assays, four replicas for RT-PCR experiments and three replicas for Western blot assays, respectively. (*p < 0.05 and **p < 0.01; between the indicated groups and ^#^*p* < 0.05 or ^##^*p* < 0^.^01 versus the CTRL group).

CCK-8 analyses revealed that KGN (0.01 μM, 0.1 μM and 1 μM) had no effect on the proliferation of chondrocytes (Fig. 1A). Moreover, KGN had no effect on mRNA expression of *Col2a1* and *Acan*, thus did not affect synthesis of cartilage matrix (Fig. 1B). Immunofluorescence staining revealed comparable effect, in which KGN had no effect on the expression of COL II protein (Fig. 1C). RT-PCR assays showed that 0.1 μM and 1 μM of KGN down-regulated the transcription levels of *Mmp13* by 13.9% and 33.6%, respectively. Similarly, 0.1 μM and 1 μM of KGN decreased the expression of *Adamts5* by 21.5% and 44.7%, respectively (Fig. 1D). Western Blot experiments confirmed that KGN down-regulated the protein levels of matrix degradation enzymes (MMP13 and ADAMTS5), whereas it barely altered the expression of matrix synthesis proteins (COL II and Aggrecan) in normal chondrocytes (Fig. 1E, F).

### KGN enhanced the synthesis of cartilage matrix in IL-1β-treated chondrocytes

**Fig. 2:**
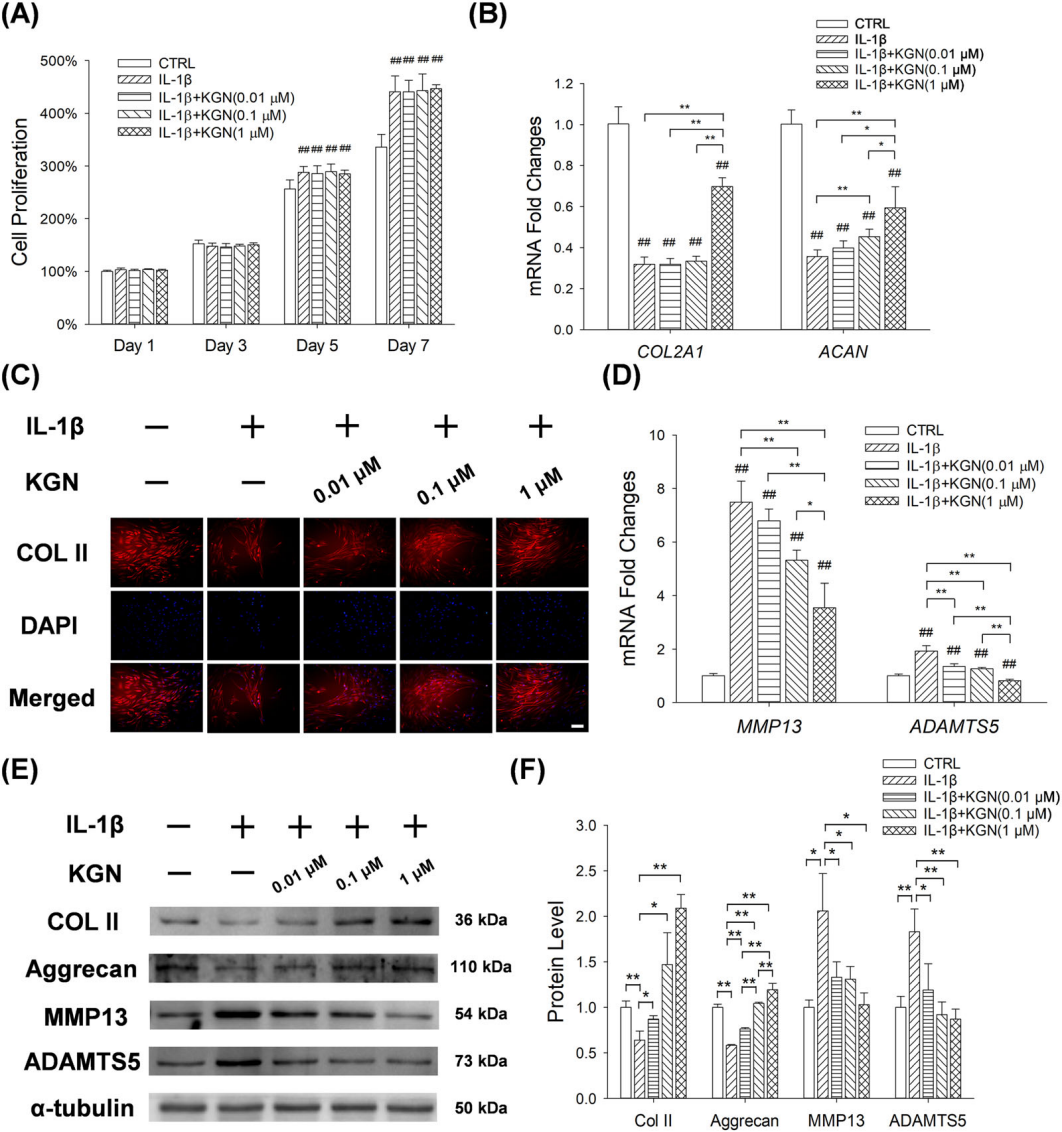
Effect of KGN on the synthesis of chondrocyte matrix after IL-1β treatment. **A** CCK-8 assay for the proliferation of chondrocytes 1, 3, 5 and 7 days after KGN treatment. **B** RT-PCR analysis for the expression levels of *COL2A1* and *ACAN* mRNA in chondrocytes after KGN treatment. **C** Immunofluorescence analysis for the expression of COL II proteins in chondrocytes after KGN treatment. (Scale bar = 100 μm). **D** RT-PCR for the expression levels of *MMP13* and *ADAMTS5* mRNA in chondrocytes after KGN treatment. **E**, **F** Western blot analysis for the expression of COL II, aggrecan, MMP13 and ADAMTS5 proteins in chondrocytes after KGN treatment. Values represent mean ± S.E.M of six replicas for cell proliferation assays, four replicas for RT-PCR experiments and three replicas for Western blot assays, respectively. (*p < 0.05 and **p < 0.01; between the indicated groups and ^#^*p* < 0.05 or ^##^*p* < 0^.^01 versus the CTRL group).

CCK-8 assay revealed that compared to controls, IL-1β treatment increased chondrocyte proliferation by 12.4% at day 5 and 17.9% at day 7. KGN treatment showed no effect on the cell proliferation of chondrocytes (Fig. 2A). RT-PCR revealed that IL-1β down-regulated the mRNA expression of *Col2A1* and *Acan* by 68.2% and 64.3%, respectively. In contrast, KGN treatment rescued the expression of cartilage matrix proteins in IL-1β-treated chondrocytes. For instance, 1 μM of KGN up-regulated transcription levels of *Col2a1* by 1.2-fold and *Acan* by 86.5% (Fig. 2B). Immunofluorescence assay validated that IL-1β inhibited the expression of COL II and KGN treatment preserved the matrix synthesis in IL-1β-treated chondrocytes (Fig. 2C). Furthermore, IL-1β remarkably increased the gene expression of *Mmp13* and *Adamts5* by 6.5-fold and 92.1%, respectively. KGN treatment significantly down-regulated the expression of these matrix degradation enzymes in a dose-dependent manner. In particular, compared with IL-1β-treated cells, 0.01, 0.1 and 1 μM of KGN decreased the mRNA levels of *Mmp13* by 9.2%, 29.0% and 52.7%, respectively. Consistently, KGN decreased the transcription levels of *Adamts5* in IL-1β-treated chondrocytes (Fig. 2D). Western Blot assays confirmed that KGN treatment up-regulated the protein expression of COL II and ACAN but down-regulated MMP13 and ADAMTS5 in the presence of IL-1β (Fig. 2E, F).

### KGN ameliorated DMM-induced OA progression in mice

**Fig. 3:**
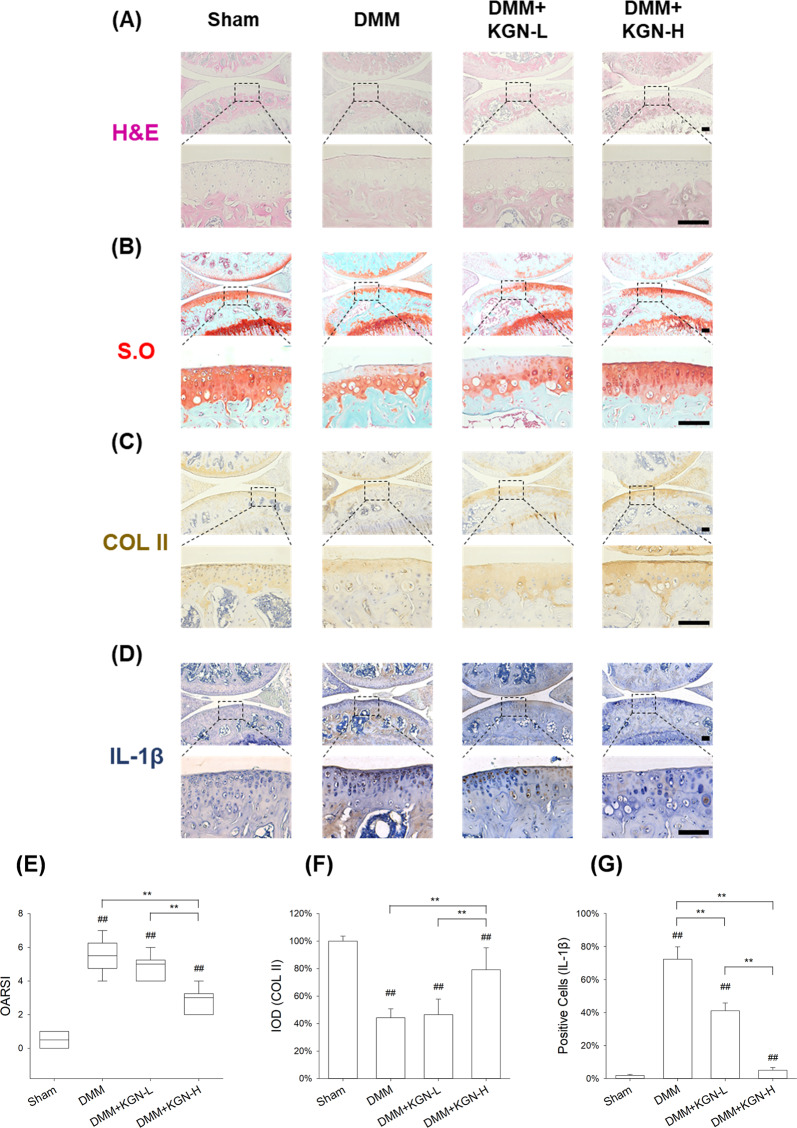
Effect of intra-articular injection of KGN on the degeneration of cartilage in DMM-induced OA in mice. DMM-op mice were injected with 1 μM or 100 μM of KGN, twice a week for four weeks, whereas the controls were injected with 10 μL of saline. **A**–**D** Representative histological images of medial femoral condyle and tibial plateau of OA mice following immunohistochemical staining. Sagittal sections of cartilage following **A** hematoxylin and eosin (H&E) staining, **B** Safranin O (S.O.)/Fast Green staining, **C** immunohistochemistry for COL II (IHC), and **D** immunohistochemistry for IL-1β (IHC). (Scale bar = 100 μm). **E** OARSI scores based on Safranin O/Fast Green staining. **F** Integrated optimal density (IOD) of COL II in articular cartilage. **G** Percentage of IL-1β positive cells in articular cartilage. In each section, the quantitative analyses were performed at three random regions, with the average used as the final value. Values represent mean ± S.E.M of six replicas. (*p < 0.05 and **p < 0.01; between the indicated groups and ^#^*p* < 0.05 or ^##^*p* < 0^.^01 versus the CTRL group).

**Fig. 4:**
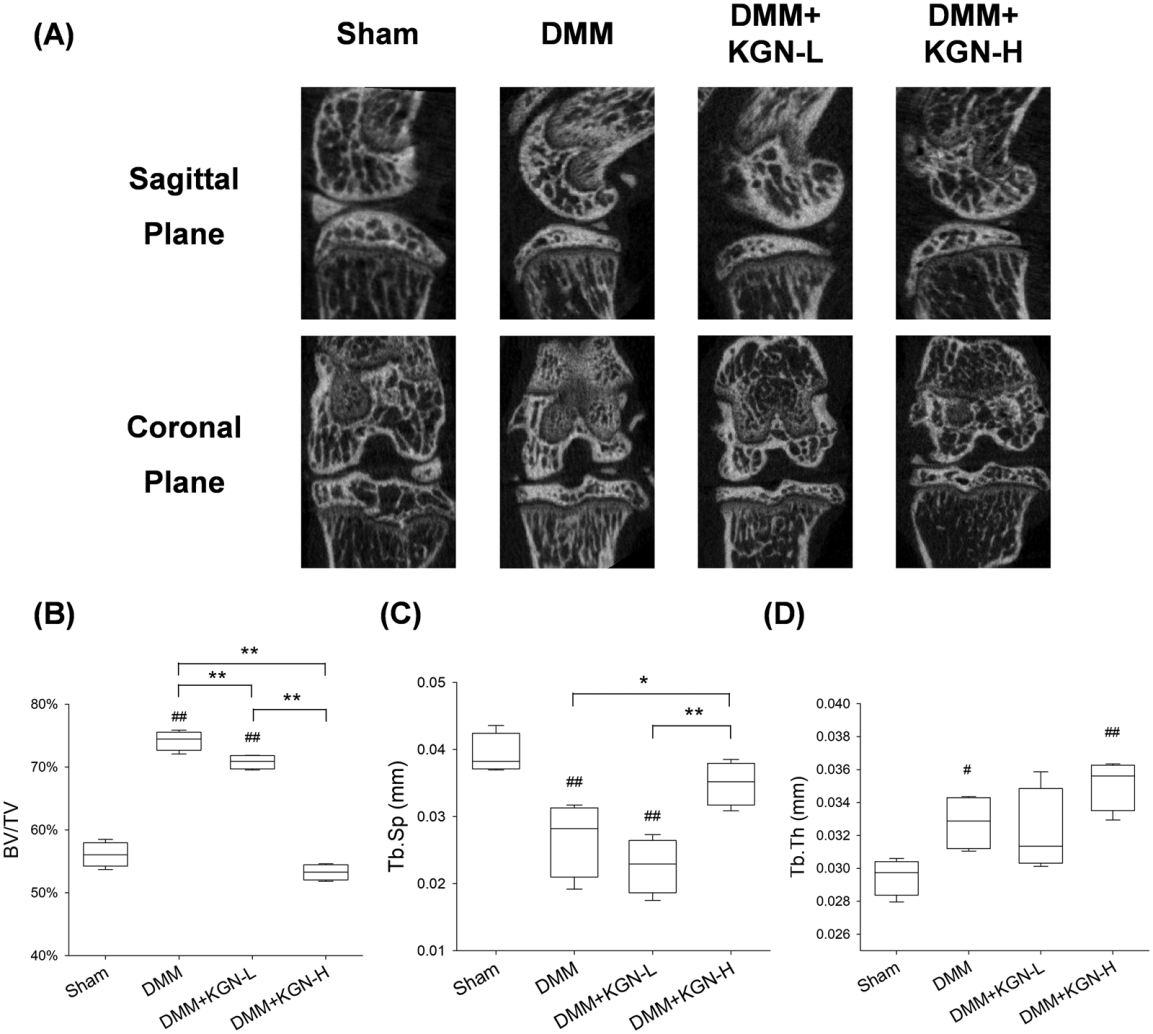
Effect of intra-articular injection of KGN on the sclerosis of subchondral bone in DMM-induced OA in mice. The DMM-op mice were injected with 1 μM or 100 μM of KGN, twice a week for four weeks, while the control groups were injected with 10 μL of saline. **A** Representative Micro-CT images of medial and coronal planes of femoral condyle and tibial plateau in mice with OA. **B**–**D** The effects of KGN on subchondral bone parameters including **B** the bone volume ratio (BV/TV, %), **C** trabecular separation (Tb.Sp., mm) and **D** trabecular thickness (Tb.Th., mm). Values represent mean ± S.E.M of four replicas. (*p < 0.05 and **p < 0.01; between the indicated groups and ^#^*p* < 0.05 or ^##^*p* < 0^.^01 versus the CTRL group).

We next investigated the effect of KGN on cartilage degeneration in experimental OA mice. KGN was intra-articular injected and H&E staining revealed KGN protected the structure of articular cartilage in OA mice (Fig. 3A). S.O. and immunohistochemistry revealed that DMM induced a marked loss of glycosaminoglycans (GAGs) and COL II in articular cartilage; however, treatment with KGN at a high concentration (100 μM) protected cartilage from OA-induced degradation (Fig. 3B, C). Furthermore, immunohistochemistry showed a strongly positive staining for IL-1β in the DMM group, whereas KGN treatment attenuated the expression of IL-1β, suggesting that KGN ameliorated the inflammatory environment induced by experimental OA (Fig. 3D). Compared to the DMM group, the OARSI grading score of the high KGN treatment group was decreased by 48.5% (Fig. 3E). Semiquantitative analysis of immunohistochemistry further confirmed that, compared to DMM group, the expression of COL II in the DMM + KGN-H group was increased by 79.3% (Fig. 3F). The percentage of IL-1β positive cells was decreased by 93.0% in the KGN-H treated group compared with the DMM group (Fig. 3G). To analyze the impact of KGN on the micro-structure of subchondral bone, μCT analysis were performed (Fig. 4A) and the results revealed that KGN-H treatment decreased the BV/TV ratio by 28.2% in DMM-op mice (Fig. 4B), while increasing Tb.Sp ratio by 30.3% (Fig. 4C). However, the Tb.Th ratio of DMM group was significantly higher than that of Sham group, which was not affected by KGN treatment (Fig. 4D). These findings suggest that KGN, particularly at high concentrations, not only protected articular cartilage from DMM-induced matrix degradation but also ameliorated the pathological sclerosis of subchondral bone.

### The effect of IL-1β on the expression of miRNAs in chondrocytes

**Fig. 5:**
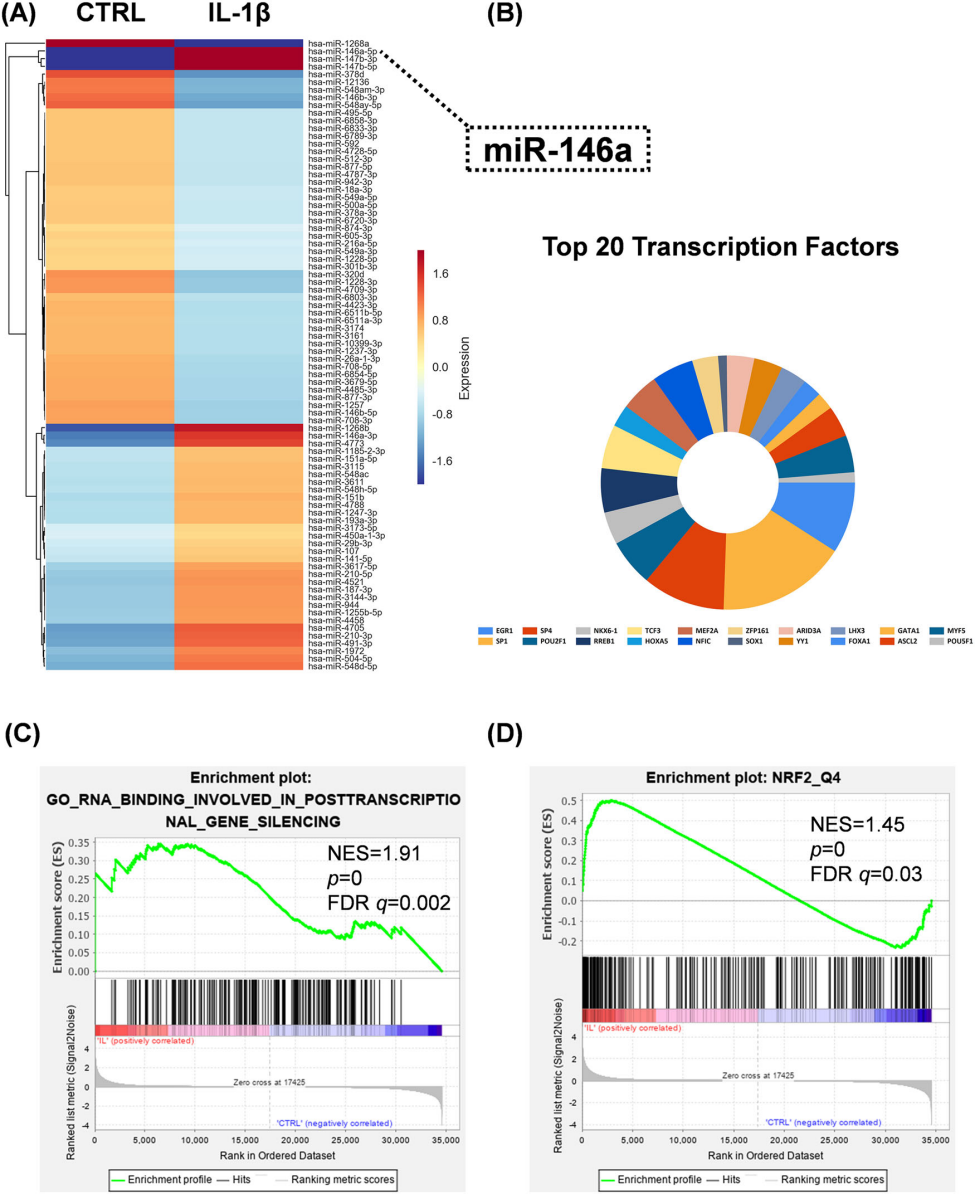
Effect of IL-1β on the expression of differentially expressed miRNAs in chondrocytes. **A** Heat map for the differentially expressed miRNAs in IL-1β-treated and control chondrocytes. The color bars on the right of the map indicate gene expression level; red denotes high expression whereas blue denotes low expression, relative to the median. **B** Enrichment analysis of differentially expressed miRNAs in IL-1β-treated and control chondrocytes. The area of the colored region corresponds to the percentage of enriched genes. **C**–**D** GSEA enrichment analysis of differentially expressed miRNAs and mRNAs in IL-1β-treated and control chondrocytes. **C** Enrichment analysis of posttranscriptional gene silencing mRNAs. **D** Enrichment analysis of NRF2 transcription factor.

To investigate the specific effect of miRNAs on IL-1β-induced cartilage matrix degradation, we performed a microarray experiment and the heat map uncovered 82 differentially expressed miRNAs in IL-1β stimulated chondrocytes (Fig. 5A). Among them, 35 miRNAs including miR-146a-5p (7.0-fold), miR-147b-3p (5.8-fold) and miR-147b-5p (6.9-fold), were up-regulated, whereas 47 miRNAs including miR-1268a (13.4-fold), miR-378d (3.1-fold) and miR-12136 (2.3-fold), were down-regulated. (Supplementary Table 2). Enrichment analysis of transcription factors revealed that Early Growth Response 1(EGR1), Sp1, Sp4, POU Class 2 Homeobox 1 (POU2F1) and NK6 Homeobox 1 (NKX6-1) were enriched in the above discrepant miRNAs (Fig. 5B). Furthermore, GSEA analysis revealed the differentially expressed miRNA and mRNA sets were associated with posttranscriptional gene silencing (Fig. 5C) and NRF2 transcription factor function (Fig. 5D). Moreover, miRNA-mRNA network of 16 differentially expressed miRNAs targeting 159 mRNAs was constructed to explore the pattern with which miRNA regulates mRNA (Supplementary Fig. 1A). Gene enrichment analysis (Supplementary Fig. 1B) revealed that 20 pathways including ECM-recetpor interaction as well as PPAR signaling, Wnt signaling, mitogen-activated protein kinase (MAPK) signaling and focal adhesion pathways were significantly up-regulated in chondrocytes. GO enrichment analysis revealed the differentially expressed miRNAs regulated several biological processes and binding of metal ions in IL-1β-stimulated chondrocytes (Supplementary Fig. 1C).

### KGN mediated anti-arthritic effects via activation of the miR-146a/NRF2 axis

**Fig. 6:**
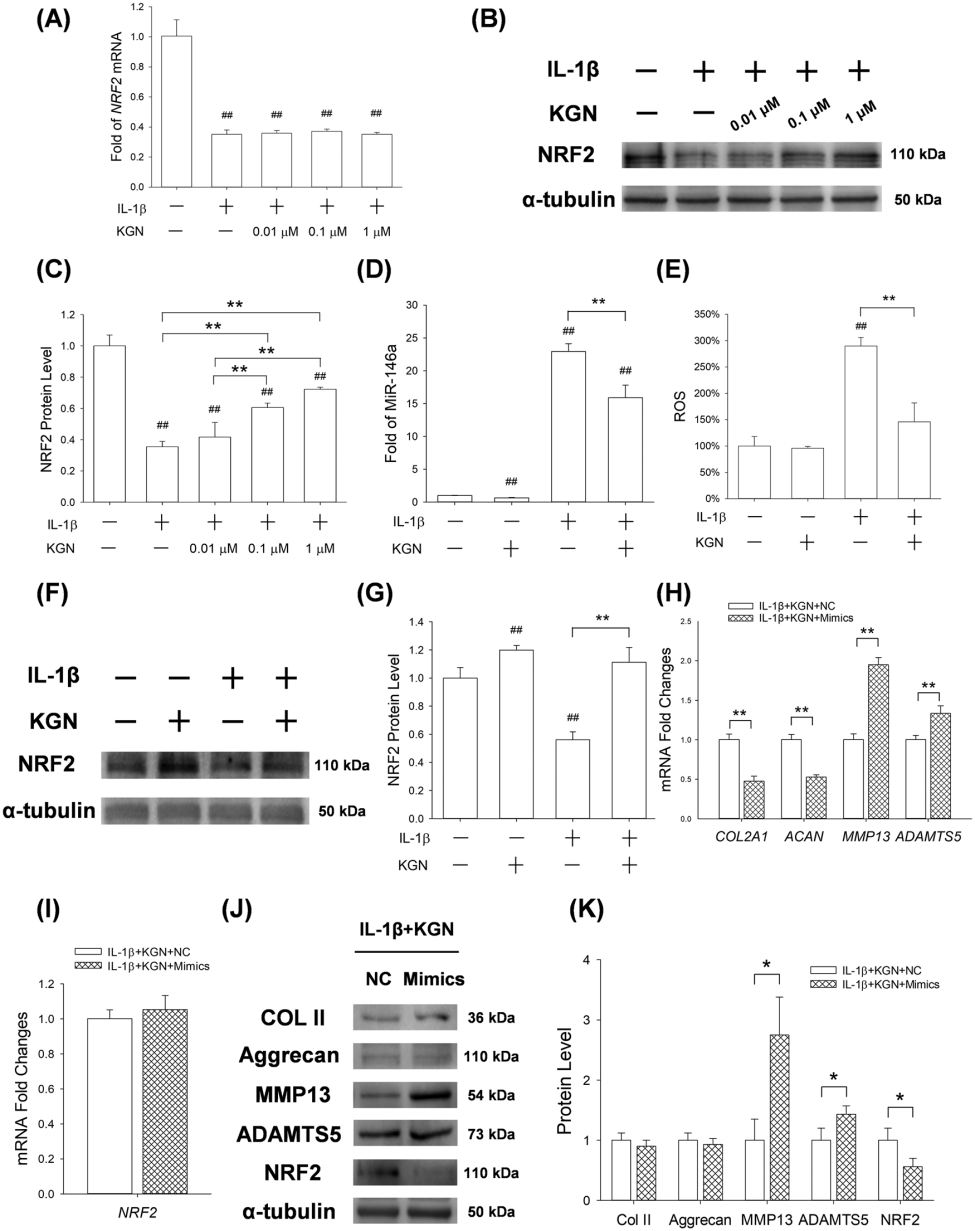
Effect of up-regulated expression of miR-146a on protective effects of KGN on cartilage matrix synthesis and the expression of NRF2 proteins in chondrocytes. Chondrocytes were treated with 5 ng/mL of IL-1β and subsequently 1 μM of KGN after transfection with miRNA-146a mimics. **A** RT-PCR analysis for the expression of *NRF2* mRNA in chondrocytes. **B**–**C** Western blot assay for the expression of NRF2 proteins in chondrocytes. **D** RT-PCR analysis for the expression of miRNA-146a in chondrocytes. (**E**) Flow cytometric analysis for the intracellular production of ROS in chondrocytes. **F**–**G** The effects of KGN on NRF2 expression in both untreated and IL-1β-treated chondrocytes were determined by Western blot assays. **H**–**I** qRT-PCR analysis for the expression of mRNAs for *COL2A1*, *ACAN*, *MMP13*, *ADAMTS5* and *NRF2*. **J**–**K** Western blot assay for the expression of COL II, aggrecan, MMP13, ADAMTS5 and NRF2 proteins in chondrocytes. Values represent mean ± S.E.M of four replicas for RT-PCR experiments and three replicas for Western blot assays, respectively. (*p < 0.05 and **p < 0.01; between the indicated groups and ^#^*p* < 0.05 or ^##^*p* < 0^.^01 versus the CTRL group).

RT-PCR analysis showed that IL-1β significantly increased the transcriptive levels of *Nrf2*. Surprisingly, KGN treatment did not reverse this phenomenon (Fig. 6A). However, Western blot assays revealed that KGN significantly increased the protein levels of NRF2 in IL-1β treated cells in a dose-dependent manner (by 17.7% at 0.01 μM, 70.7% at 0.1 μM and 103.9% at 1 μM; Fig. 6B, C), suggesting that KGN may affect the expression of NRF2 in a post-transcriptional manner. RT-PCR assays further revealed that the expression of miR-146a in IL-1β-treated chondrocytes was 21.9-fold higher than in controls, but underwent a 30.7% down-regulation after treatment with 1 μM of KGN (Fig. 6D). KGN treatment also significantly modulated ROS production in IL-1β-stimulated chondrocytes (Fig. 6E). Furthermore, Western Blot assay showed that KGN increased the expression of NRF2 by 19.8% and 98.2% in untreated and IL-1β-treated chondrocytes, respectively (Fig. 6F, G).

To explore the role of miR-146a in KGN-mediated anti-arthritic effects, chondrocytes were transfected with miR-146a mimics before IL-1β and KGN treatment (Supplementary Fig. 2A). RT-PCR results showed that over-expression of miR-146a decreased the mRNA expression of *Col2a1* and *Acan* by 52.5% and 47.3%, respectively. Meanwhile, over-expression of miR-146a up-regulated the transcript levels of *Mmp13* and *Adamts5* in KGN-treated chondrocytes by 94.6% and 33.3%, respectively (Fig. 6H). However, over-expression of miR-146a did not affect the gene expression of *Nrf2* (Fig. 6I). Western blot assays validated the effects of miR-146a over-expression on the synthesis and degradation of cartilage matrix. In particular, miR-146a decreased the protein expression of NRF2 in chondrocytes by 43.6% (Fig. 6J, K). In the absence of IL-1β or KGN, over-expression of miR-146a exhibited similar effects on the expression of matrix proteins and degradation enzymes (Supplementary Fig. 2B–F).

### Overexpression of miR-146a abolished protective effects of KGN on cartilage matrix

**Fig. 7:**
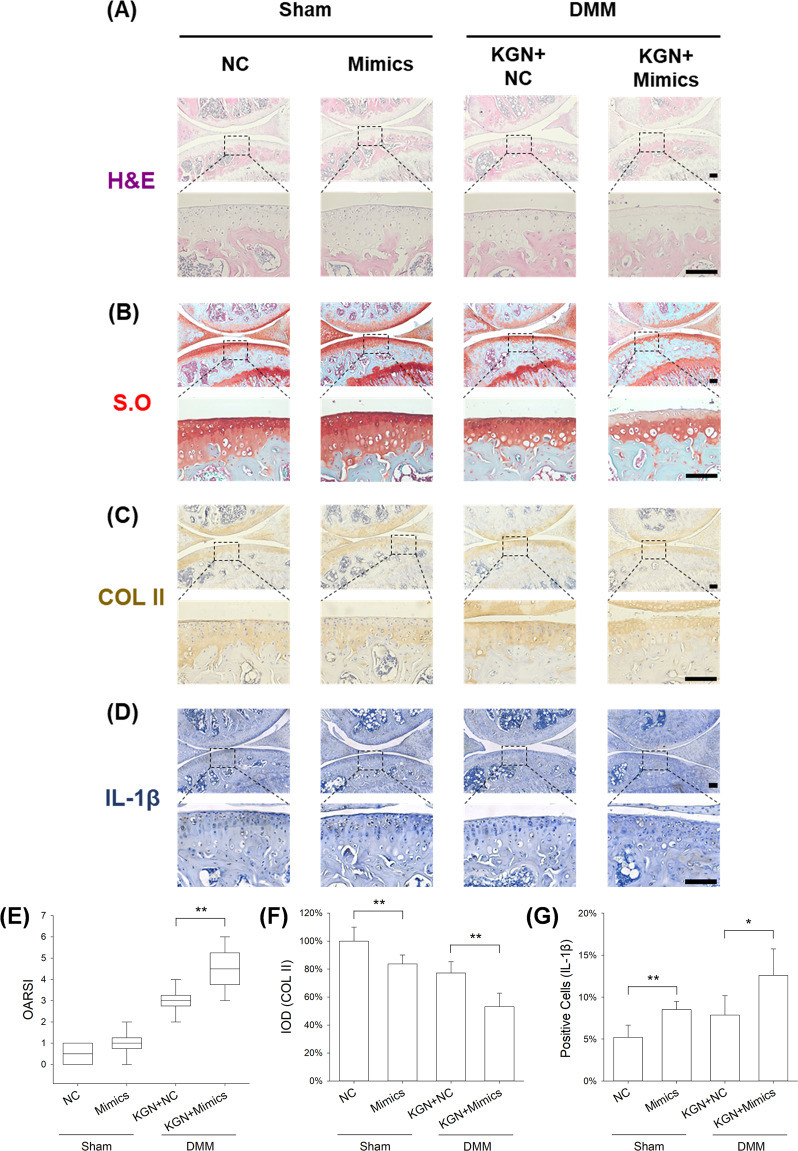
Effect of up-regulated expression of miR-146a on KGN protective function against cartilage degeneration. **A**–**D** Representative histological images of medial femoral condyle and tibial plateau of OA mice following immunohistochemical staining. Sagittal sections of cartilage following **A** hematoxylin and eosin (H&E) staining, **B** Safranin O (S.O.)/Fast Green staining, **C** immunohistochemistry for COL II (IHC), and **D** immunohistochemistry for IL-1β (IHC). (Scale bar = 100 μm). **E** OARSI scores based on Safranin O/Fast Green staining. **F** Integrated optimal density (IOD) of COL II in articular cartilage. **G** Percentage of IL-1β positive cells in articular cartilage. In each section, the quantitative analyses were performed at three random regions, with the average used as the final value. Values represent mean ± S.E.M of six replicas. (*p < 0.05 and **p < 0.01; between the indicated groups and ^#^*p* < 0.05 or ^##^*p* < 0^.^01 versus the CTRL group).

**Fig. 8:**
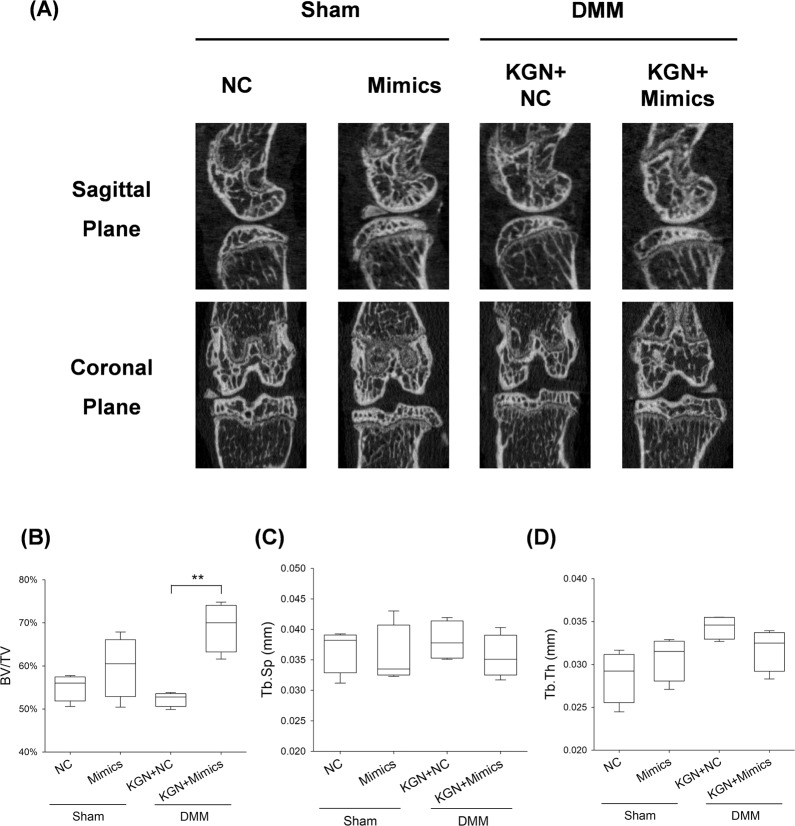
Effect of up-regulated expression of miR-146a on protective effect of KGN -against subchondral bone sclerosis. **A** Representative Micro-CT images for medial and coronal planes of femoral condyle and tibial plateau in OA mice. **B**–**D** The effects of KGN on subchondral bone parameters including **B** bone volume ratio (BV/TV, %), **C** trabecular separation (Tb.Sp., mm), and **D** trabecular thickness (Tb.Th., mm). Values represent mean ± S.E.M of four replicas. (*p < 0.05 and **p < 0.01; between the indicated groups and ^#^*p* < 0.05 or ^##^*p* < 0^.^01 versus the CTRL group).

We further investigated the role of miR-146a in modulating OA-induced cartilage degeneration. We found intra-articular injection of miR-146a had no effect on the structure of articular cartilage or expression of matrix proteins in sham-op mice. However, in DMM-op mice, treatment with miR-146a mimics counteracted the protective effects of KGN on cartilage matrix. In particular, miR-146a suppressed the expression of GAGs and COL II while increasing the level of IL-1β (Fig. 7A–D). Compared to KGN-treated OA mice, the OARSI score was increased by 50.0%, the IOD of COL II was decreased by 31.1%, and the percentage of IL-1β positive cells was increased by 60.3% in miR-146a mimics group (Fig. 7E–G). The results of μCT analysis confirmed that treatment with miR-146a mimics aggravated subchondral bone sclerosis in DMM mice (Fig. 8A). The ratio of BV/TV was significantly increased by 32.1% in the miR-146a mimics group (Fig. 8B), but the values of Tb.Sp and Tb.Th were not significantly different between the two groups (Fig. 8C, D).

## Discussion

Increasing evidence has indicated that KGN is able to protect articular cartilage from OA-induced degeneration^20,21^. For instance, Fan et al. demonstrated that intra-articular injection of KGN conjugated polyurethane nanoparticles relieved the progression of post-traumatic OA in mice^22^. Consistently, we found that KGN treatment ameliorated articular cartilage deterioration in an experimental OA mouse model by inhibiting matrix degradation and preventing abnormal sclerosis of the subchondral bone. Cartilage degeneration and subchondral bone resorption are typical markers of early OA, while appearance of osteophytes with elevated bone formation is the hallmark in the late stage of OA^23^. In this study, we observed a significant increase in BV/TV ratio in subchondral bone of DMM-op mice. KGN treatment suppressed aberrant osteogenesis in mice OA. Similarly, Klose-Jensen et al. reported that patients with end-stage hip OA were presented with higher BV/TV in the subarticular region, which was correlated with the degree of local cartilage degeneration^24^. Therefore, these findings suggested that KGN can potentially prevent OA-induced abnormal subchondral bone formation; however, the underlying mechanisms remain to be further investigated. Sclerostin plays a critical role in promoting formation of subchondral bone during OA progression, since a drastic reduction of sclerostin expression was detected in osteocytes and knockout of sclerostin did not induce the increase in the thickness of the subchondral bone plate^25^.

In vitro studies revealed that KGN preserved the expression of COL II and aggrecan in IL-1β-treated chondrocytes, the two major anabolic components in cartilage matrix. Notably, KGN treatment markedly down-regulated the expression of MMP13 and ADAMTS5 in both IL-1β-stimulated and normal chondrocytes. MMP13 and ADAMTS5 induce degradation of ECM cartilage by hydrolyzing network of collagen fibers and proteoglycan structure^26^. Wang et al. demonstrated that intra-articular injection of KGN-incorporated thermogel inhibited joint inflammation in rabbits with OA by down-regulating the expression of MMP13^27^. KGN inhibited the expression of MMP13 possibly by enhancing the phosphorylation of c-Jun N-terminal kinase (JNK) and subsequently suppressing the β-catenin pathway^28^. It is worth noting that KGN exerted an inhibitory effect on matrix degradation rather than matrix synthesis in normal cultured chondrocytes. We considered that this difference may be attributed to the metabolic characteristic of mature chondrocytes, since chondrocytes cultured in the normal condition had a strong potential to produce matrix components. Unlike chondrocytes, the chondrogenic differentiation of mesenchymal stem cells and synthesis of cartilage matrix components can be remarkably enhanced by KGN^29^. KGN was hard to further increase the high level of matrix synthesis in mature chondrocytes, but when exposed to an inflammatory environment, KGN had the potential to improve the low level of matrix synthesis that was inhibited by IL-1β. However, we found that KGN protected the expression of cartilage matrix components such as Collagen II and aggrecan in IL-1β-stimulated chondrocytes, suggesting that KGN had the potential to promote cartilage matrix synthesis in an inflammatory environment.

As a crucial endogenous pro-inflammatory cytokine, IL-1β not only mediates the inflammatory response in cooperation with other cytokines such as inducible nitric oxide synthase (iNOS), IL-6, and tumor necrosis factor-α (TNF-α), but also aggregates the matrix degradation via activation of the nuclear factor-kappa B (NF-κB) pathway^30^. In this study, we showed that the anti-arthritic effects of KGN protected articular chondrocytes from in vitro IL-1β stimulation. Consistently, the IHC results confirmed the anti-inflammatory effects of KGN on OA cartilage, evidenced by the fact that KGN treatment attenuated the expression of IL-1β in DMM-induced degenerative articular cartilage. In addition, TNF-α, as another important inflammatory cytokine, also plays an important role in OA pathogenesis by promoting the production of MMPs to exacerbate the cartilage destruction through the NF-κB pathway^31^. Furthermore, TNF-α has been shown to induce chronic inflammation in cartilage and subchondral bone that delayed the self-repairing at the damaged area^32^. Hong et al. found that administration of melatonin in rats reduced the concentration of IL-6 and TNF-α in early OA, indicating a protective role of melatonin in encountering inflammation in vivo^33^. However, the effects of KGN on protecting articular cartilage from TNF-α-induced degeneration are still unknown and will be investigated in our future studies.

To unravel the underlying mechanisms of KGN-mediated protection against OA, we found that KGN inhibited ROS accumulation and maintained redox balance in OA chondrocytes via the NRF2-medicated antioxidant pathway. Pan et al. previously suggested that activation of NRF2 by myricetin not only inhibited the production of iNOS and cyclooxygenase-2 (COX-2) in IL-1β-treated chondrocytes, but also attenuated cartilage destruction by suppressing MMP13 and ADAMTS5^34^. Meanwhile, Kruppel-like factor 2 (KLF2), a member of the zinc finger family, also participates in the antioxidant response in OA chondrocytes. In particular, KLF2 promotes the nuclear translocation of NRF2 and enhances the transcription of down-stream antioxidant enzymes such as NAD (P) H dehydrogenase quinone 1 (NQO1)^35^. Activation of NRF2 has been shown to up-regulate the expression of HO-1, which enhances ECM synthesis and inhibits chondrocyte apoptosis^36^. Cai et al. demonstrated that *Nrf2* knockout in mice exacerbated articular cartilage erosion after DMM surgery, suggesting the critical role NRF2 in maintaining cartilage homeostasis^37^.

Interestingly, in this study, we found that KGN treatment did not affect the transcription levels of *Nrf2*, while significantly up-regulating its protein expression. Thus, we speculated that KGN might regulate the expression of NRF2 in OA chondrocytes at the post-transcriptional level. However, the precise mechanism involves in the regulation of miR-146a on NRF2 remains unknown. Smith et al. reported that transfection with a miRNA-146a mimic caused a marked reduction in *Nrf2* translation in young rat hepatocytes by directly binding to *Nrf2* mRNA and suppressed its translational process^17^. In addition, miR-142 was shown to inhibit the gene expression of *Nrf2* in esophageal squamous cell carcinoma (ESCC) cells, suggesting that the microRNAs may regulate the transcription process of *Nrf2* gene or induce its mRNA degradation^38^. To elucidate the crosstalk between miR-146a and NRF2, both microarray analysis and real-time RT-PCR were performed and implicated the role of miR-146a in regulating the protein expression of NRF2 in KGN-treated chondrocytes. Further experiments validated this finding, in which over-expression of miR-146a not only decreased the expression of NRF2, but also abolished the protective effects of KGN on cartilage matrix degeneration. Consistently, Guan et al. reported that miR-146a modulated cartilage degeneration in aging-associated and trauma-induced OA. Knockout of miR-146a in mice triggered the early onset of OA and exacerbated post OA cartilage degeneration^39^. However, the role of miR-146a in the development of OA is still controversial. For instance, Yamasaki et al. reported that the expression of miR-146a was at a high level in joint cartilage of patients with mild OA, whereas it was decreased in the severe OA cases, indicating the dynamic change of miR-146a in OA cartilage^40^. Therefore, we speculated that miR-146a is an oxidative stress-sensitive microRNA^18^ that can be up-regulated in response to mechanical injury by a compensatory mechanism. Nonetheless, the role of miR-146a in OA development needs further investigation.

It is unclear how KGN regulated the expression of miR-146a in OA chondrocytes. KEGG enrichment analysis revealed that the MAPK pathway was activated in IL-1β-stimulated chondrocytes. In a recent study, Jing et al. reported that KGN enhanced the phosphorylation of (c-Jun N-terminal kinase) JNK, an important kinase in the MAPK signaling pathway that promoted the deposition of cartilage matrix in human umbilical cord MSCs^28^. This finding implied that KGN might regulate the miR-146a-NRF2 axis through the MAPK signaling pathway. In addition, long non-coding RNA (lncRNA) such as lncRNA X inactivate-specific transcript (XIST)^41^, lncRNA metastasis-associated lung adenocarcinoma transcript 1 (MALAT1)^42^ and lncRNA cardiac hypertrophy-related factor (CHRF)^43^, have been reported to regulate the expression of miR-146a. Xi et al. demonstrated that lncRNA HCG18 as an endogenous competitor RNA (ceRNA) was able to inhibit the expression of miR-146a. This resulted in intervertebral disc degeneration by disrupting the TNF receptor associated factor 6 (TRAF6)/NF-κB pathway^44^. In our future studies, the precise mechanisms of KGN-mediated regulation of miR-146a will be further explored.

## Conclusions

We demonstrated that KGN promoted the synthesis of cartilage matrix and suppressed the expression of matrix degrading enzymes in IL-1β-stimulated chondrocytes. In an experimental OA mouse model, intra-articular injection of KGN protected against articular cartilage degeneration and aberrant subchondral bone formation. Mechanistically, KGN up-regulated the protein expression of NRF2 by down-regulating miR-146a at a post-transcriptional level. Over-expression of miR-146a abolished the protective effects of KGN on chondrocytes against IL-1β stimulation and aggravated the cartilage degeneration in surgically induced OA mice. In our future studies, we will investigate the role of the KGN-mediated miR-146a-NRF2 axis in articular cartilage and subchondral bone degradation in the late stage of OA.

## Materials and methods

### Isolation and culture of human chondrocytes

The protocol for this study was approved by the Ethic Committee of The First Affiliated Hospital of Soochow University. Human cartilage samples from the femoral condyle and tibial plateau were obtained from 6 patients (3 females and 3 males with a mean age of 52 years) who underwent total knee arthroplasty surgery at the hospital. Cartilage tissues were cut into small pieces of 1 mm^3^ each before overnight digestion at 37 °C with 0.2% type II collagenase (Thermo Fisher Scientific, Waltham, MA, USA). They were washed three times with phosphate buffer saline (PBS) and cultured in a mixture of Dulbecco’s Modified Eagle Medium and F-12 nutrient (DMEM/F12) supplemented with 10% fetal bovine serum (FBS), 100 units/ml penicillin, and 100 mg/ml streptomycin (Thermo Fisher Scientific). The culture medium was changed every three days, with passage one (P1) chondrocytes used in the subsequent experiments.

### Effects of In vitro treatment with IL-1β, KGN, and miR-146a mimics

To establish an inflammatory OA environment in vitro, cultured chondrocytes were treated with 5 ng/ml of recombinant human interleukin-1 beta (IL-1β) (Peprotech, Rocky Hill, NJ, USA)^45^. KGN (Sigma-Aldrich, St. Louis, MO, USA) was first dissolved in dimethyl sulfoxide (DMSO) to a stock concentration of 20 mM before further dilution with PBS. Chondrocytes were treated with serial concentration of KGN (0.01 μM, 0.1 μM or 1 μM), whereas the control group was treated with 0.005% DMSO. Chondrocytes were seeded in 6-well plates and thereafter transfected with miR-146a mimics (100 nM, GenePharma, Shanghai, China) or controls (NC, 100 nM) via Lipofectamine 2000 (Thermo Fisher Scientific), according to the manufacture’s protocol. The sequence of miR-146a mimics was 5′-UGAGAACUGAAUUCCAUGGGUU-3′ and 5′-CCCAUGGAAUUCAGUUCUCAUU-3′ for negative control (NC).

### Cell proliferation

Cell Counting Kit-8 assays (CCK-8, Beyotime Institute of Biotechnology, Haimen, China) were performed as previously described^46^. Briefly, chondrocytes (1 × 10^3^ cells/well) were first seeded in 96-well plates before treatment with IL-1β or KGN. At days 1, 3, 5 and 7, the cells were incubated for 1 h at 37 °C in 10% CCK-8 solution. The optical density (OD) of the cell culture was measured at 450 nm using a microplate spectrophotometer (BioTek, Winooski, VT, USA).

### Immunofluorescence

Chondrocytes in 24-well plates were fixed for 15 min in 4% paraformaldehyde and permeabilized for 10 min using 0.1% Triton X-100 (Sigma-Aldrich). The cells were blocked for 30 min in 1% bovine serum and thereafter incubated for 1 h in a properly diluted anti-COL II (1:200, ab34712, Abcam, Cambridge, MA, USA) primary antibodies. After three washes with PBS, the cells were incubated for 1 h with Alexa Fluor® 647 conjugated secondary antibodies (ab150075, Abcam). The nucleus of the cells was counterstained using 4’,6-diamidino-2-phenylindole (DAPI, Thermo Fisher Scientific). Images of the cells were captured using a Zeiss Axiovert 40CFL microscope (Zeiss, Oberkochen, Germany).

### Quantitative real-time reverse transcription-polymerase chain reaction (RT-PCR)

Total RNA of the chondrocytes was extracted using TRIzol^®^ (Thermo Fisher Scientific). Complementary DNA (cDNA) synthesis of 1 μg the RNA was performed using the RevertAid First Strand cDNA Synthesis Kit (Thermo Fisher Scientific). RT-PCR amplification of the cDNA was performed using an iTap™ Universal SYBR^®^ Green Supermix kit (Bio-Rad, Hercules, CA, USA) in an CFX96™ Real-Time PCR System (Bio-Rad). Transcriptional levels of *COL2A1* (type II collagen), *ACAN* (aggrecan), *MMP13*, *ADAMTS5* and *NRF2* were then determined, in which *GAPDH* (glyceraldehyde 3-phosphate dehydrogenase) was used as the standard control. The relative expression level of each target gene was calculated based on the formulae Ct (2^−ΔΔCt^). Meanwhile, the expression of miR-146a-5p and RNU6-6P (RNA, U6 small nuclear 6) was evaluated using the TaqManTM Fast Advanced Master Mix (Thermo Fisher Scientific). The Primer sequences for the target genes are listed in Supplementary Table 1.

### Production of Intracellular reactive oxygen species (ROS) test

Here, after 24 h treatment with IL-1β and KGN, the chondrocytes were incubated for 10 min at 37 °C in 10 μM of 2′,7′-dichlorofluorescein diacetate (DCFH-DA, Beyotime). Fluorescence intensity of cells (10,000) in each group was measured using a Guava EasyCyte Flow Cytometer (FCM, Millipore, Boston, MA, USA) and analyzed using FlowJo 10.7 software (TreeStar, San Carlos, CA, USA).

### Western blotting

Total proteins in the chondrocytes were extracted using radioimmunoprecipitation assay (RIPA) lysis buffer containing protease inhibitors. The concentration of different proteins was measured using the BCA Protein Assay Kit (Beyotime), following the manufacturers protocol. Equal amounts of protein were then separated by electrophoresis in 10% sodium dodecyl sulfate-polyarylamide gel (SDS-PAGE) and subsequently transferred to nitrocellulose membranes. The membranes were then blocked using a blocking buffer (Beyotime) and thereafter incubated overnight at 4 °C with several antibody solutions; anti-COL II (ab34712), anti-aggrecan (ab3778), anti-MMP13 (ab39012), anti-ADAMTS5 (ab41037), anti-NRF2 (ab137550) or anti-α-tubulin (ab7291). Second incubation was performed for 1 h at room temperature with secondary antibodies (Abcam). The membranes were incubated in Chemiluminescence solution (SuperSignal West Pico Substrate, Thermo Fisher Scientific) to enhance visualization of the proteins. The proteins were quantifies based on the intensity of blot bands using ImageJ software (National Institutes of Health, Bethesda, MD, USA).

### Microarray analysis for microRNA expression

Microarray analysis was performed using the Affymetrix Gene Chip miRNA 2.0 arrays (Affymetrix, Santa Clara, CA, USA), with quality control performed using Affymetrix miRNA QC Tool. The expression intensities of miRNA transcripts were calculated using Affymetrix Gene Chip Command Console v.3.2. Microarray hybridization, scanning and analysis were performed at Shanghai OE Biotech. Co., Ltd. (Shanghai, China). Differentially expressed miRNAs with a fold change > 1.5 were exported to a heatmap using the Agilent Feature Extraction software (version 11.0.11, Agilent Technologies). The associated differently expressed genes and corresponding pathways were predicted using the Targetscan (www.Targetscan.org) and miRDB (http://mirdb.org) online platforms. Differentially expressed mRNA array (GSE75181) was downloaded from Gene Expression Omnibus (GEO) database (www.ncbi.nlm.nih.gov/gds/). The differently expressed mRNA in this study were then analyzed using Gene set enrichment analysis (GSEA) software (http://www.broad.mit.edu/GSEA, v.4.0.3), based on online platforms including Reactome gene sets and transcription factor targets.

### Mouse model of surgically induced OA

Protocols for animal experiments were approved by the Ethics Committee of The First Affiliated Hospital of Soochow University. C57BL/6 J mice (6~8 weeks old, male) were purchased from The Experimental Animal Center of Soochow University. The knee OA model was established by surgical destabilization of medial meniscus (DMM)^47^. Briefly, the mice were first anesthetized with a mixture of 2.0% isoflurane and 30% oxygen (RWD Life Science, Shenzhen, China). The medial meniscotibial ligaments (MML) of OA group were then transected using microscissors. For sham groups, the knee capsules were only exposed but the integrity of MMLs were not interfered with. All surgeries were performed by the same surgeon (ZY).

### Intra-articular injection of KGN and the miRNA-146a mimics

KGN stock solution was dissolved in DMSO and then diluted in 0.9% (wt/vol) sterile saline solution. Ten microliters of saline or KGN (1 μM or 100 μM) solution were injected into the knee joint using a Hamilton micro syringe. Intra-articular injections were performed twice a week for 4 weeks after euthanasia. For the effect of miRNA-146a on OA, 10 μl of NC or miR-146a mimics (250 μM) solution mixed with KGN were administered to the mice via intra-articular injection.

### Histology and immunohistochemistry

Knee joint specimens were extracted from the mice, fixed for 48 h in 10% formalin and decalcified for 14 days in 10% EDTA (pH = 7.4, Sigma-Aldrich). The samples were then embedded in paraffin and cut in 6 µm thick section, which were stained with hematoxylin and eosin (H&E) and Safranin O (S.O.)/Fast Green (Sigma-Aldrich). Images of the tissues were captured using a bright-field microscope (Zeiss Axiovert 200, Oberkochen, Germany). Histological scores of the sections were calculated independently by three researchers. The degree of cartilage degeneration was graded according to the Osteoarthritis Research Society International (OARSI) scoring system^48^.

For immunohistochemistry (IHC) experiment, sections were deparaffinated in xylene and dehydrated in serial dilutions of analytical grad alcohol. After incubation in 1% hydrogen peroxide for 30 min, the tissue sections were further incubated for 30 min in 2 mg/mL of testicular hyaluronidase (Sigma-Aldrich) for 30 min at 37 °C. They were then blocked for 30 min using 1.5% goat serum, and thereafter incubated overnight at 4 °C in specific anti-COL II (ab34712, Abcam) or anti-IL-1β (16806-1-AP, Proteintech) primary antibodies. The tissues were further incubated for 30 min in biotinylated goat anti-rabbit secondary antibodies (Vector Laboratories, Burlingame, CA, USA), before signal amplification using Vectastain Elite ABC kit (Vector Laboratories). The staining was performed using 3,3′-diaminobenzidine solution (DAB, Vector Laboratories), whereas nucleus counterstaining was performed using hematoxylin. Integrated optimal density (IOD) of COL II were semi-quantified using Image-Pro Plus (IPP) software, v.6.0 (Media Cybernetics, Bethesda,MD, USA) and the percentage of IL-1β-positive cells was counted (per 100 μm^2^ area in each section) for quantitative evaluation.

### Micro-computed tomography (μCT) analysis

μCT analysis was performed using the Skyscan-1176 scanning system (Kontich, Belgium) as previously described, under 50 kV/200 μA and 9 µm per pixel^49^. Data reconstruction was performed using NRecon v1.6 workstation and CTAn v1.13.8.1 software. The subchondral bone at the medial tibial plateau was the region of interest (ROI) for the quantitative analysis. The parameters of interest included the bone volume fraction (BV/TV, %), trabecular thickness (Tb.Th., mm) and trabecular separation (Tb.Sp., mm), which were indicative of the severity of sclerosis of the subchondral bone.

### Statistical analysis

Continuous data were presented as means of at least three independent experiments ± standard error of mean (S.E.M.). Differences among groups were evaluated using one-way or a two-way Analysis of Variance (ANOVA) and Tukey’s post hoc test. Student’s *t*-test was used to perform two-group comparisons. *P < 0.05 or **P < 0.01 was considered statistically significant. All statistical analyzes were performed using SPSS software version 13.0 (SPSS Inc., Chicago, IL, USA).

## Supplementary information

Supplementary Figures

Supplementary Tables
