# Supplementary material for: Kartogenin prevents cartilage degradation and alleviates osteoarthritis progression in mice via the miR-146a/NRF2 axis
Source: Cell Death Dis. 2021 May 13;12(5):483. doi: 10.1038/s41419-021-03765-x (PMC8119954; doi:10.1038/s41419-021-03765-x)
Supplement: Supplementary file 1 — Supplementary Figures [file 41419_2021_3765_MOESM1_ESM.docx]

**Supplementary Figures and Figure legends**


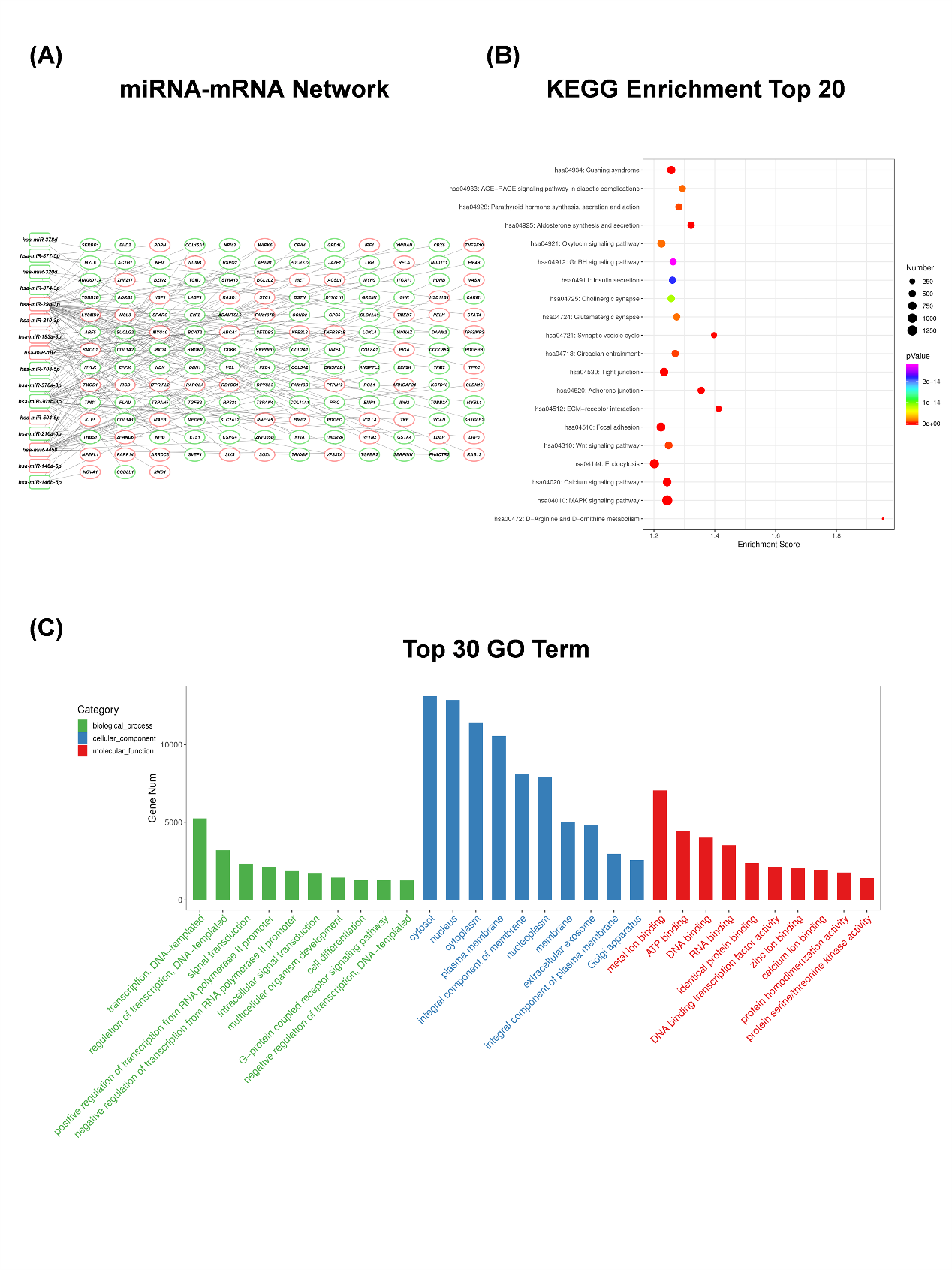


**Supplementary Figure 1.** Enrichment analysis for the differentially expressed miRNAs in IL-1β stimulated chondrocytes. (A) The miRNA-mRNA network of 16 miRNAs and 159 mRNAs differentially expressed in IL-1β stimulated chondrocytes. (B) Pathway enrichment analysis of miRNA targets. The size of the dots (gene count) corresponds to the number of genes (predicted targets of differentially expressed miRNAs) involved in each biological process. The color of the dots represents p value. (C) Gene Ontology (GO) enrichment analysis for miRNA targets. GO assessed the biological processes (BP), cellular component (CC), and molecular function (MF) of the miRNA target genes.


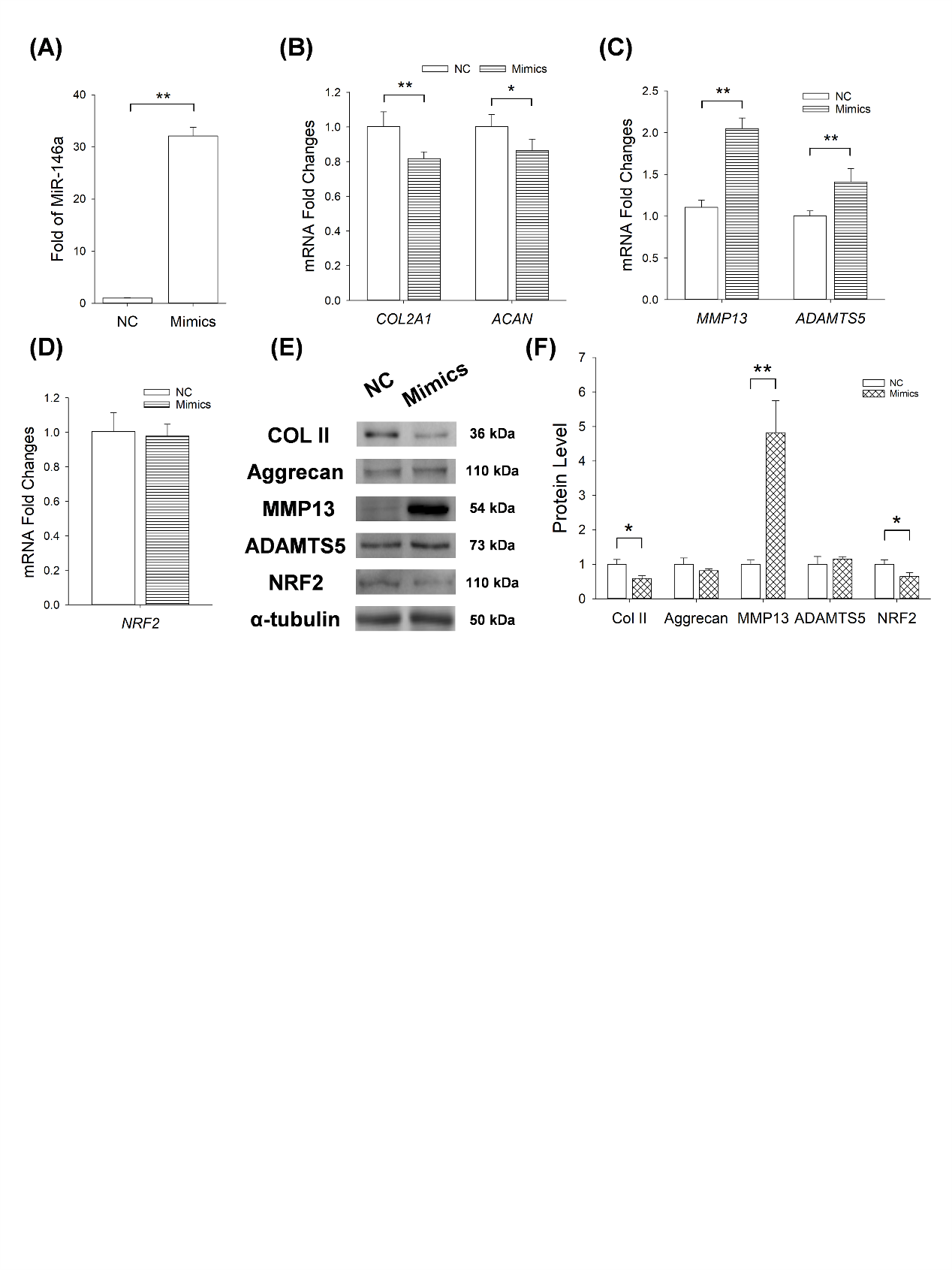


**Supplementary Figure 2.** Effect of up-regulated expression of miR-146a on treating naive chondrocytes. (A) qRT-PCR for the expression of miRNA-146a. (B-D) qRT-PCR for the expression levels of (B) *COL2A1* and *ACAN*, (C) *MMP13* and *ADAMTS5* mRNA and (D) *NRF2.* (E-F) Western blot assay for the expression of (E) anabolism, catabolism and NRF2 proteins and (F) quantification of the proteins. Values represent mean ± S.E.M of four replicas for RT-PCR experiments and three replicas for Western blot assays, respectively. (* *p* < 0.05 and ** *p* < 0.01; between the indicated groups and ^#^ *p* < 0.05 or ^##^ *p* < 0.01 versus the CTRL group).
