# Supplementary material for: Kartogenin prevents cartilage degradation and alleviates osteoarthritis progression in mice via the miR-146a/NRF2 axis
Source: Cell Death Dis. 2021 May 13;12(5):483. doi: 10.1038/s41419-021-03765-x (PMC8119954; doi:10.1038/s41419-021-03765-x)
Supplement: Supplementary file 2 — Supplementary Tables [file 41419_2021_3765_MOESM2_ESM.docx]

**Supplementary Table 1.** Primers used for real-time PCR

| **Gene** | **Forward Primer sequence (5’-3’)** | **Reverse Primer sequence (5’-3’)** |
| --- | --- | --- |
| *COL2A1* | TGGACGCCATGAAGGTTTTCT | TGGGAGCCAGATTGTCATCTC |
| *ACAN* | ACTCTGGGTTTTCGTGACTCT | ACACTCAGCGAGTTGTCATGG |
| *MMP13* | ACTGAGAGGCTCCGAGAAATG | GAACCCCGCATCTTGGCTT |
| *ADAMTS5* | ACTACGATGCAGCTATCCTGT | GTCCCAACGTCTGCCATTC |
| *NRF2* | TCAGCGACGGAAAGAGTATGA | CCACTGGTTTCTGACTGGATGT |
| *GAPDH* | AGAAAAACCTGCCAAATATGATGAC | TGGGTGTCGCTGTTGAAGTC |

**Supplementary Table 2.** MicroRNA expression profiles of human articular chondrocytes in response to IL-1β stimuli.

| *No* | *microRNA* | *Fold Change* | *Regulation* |
| --- | --- | --- | --- |
| 1 | hsa-miR-146a-3p | 9.55 | Up |
| 2 | hsa-miR-491-3p | 8.84 | Up |
| 3 | hsa-miR-1972 | 8.35 | Up |
| 4 | hsa-miR-3144-3p | 8.03 | Up |
| 5 | hsa-miR-3115 | 7.35 | Up |
| 6 | hsa-miR-548ac | 7.35 | Up |
| 7 | hsa-miR-146a-5p | 6.98 | Up |
| 8 | hsa-miR-147b-5p | 6.94 | Up |
| 9 | hsa-miR-147b-3p | 5.78 | Up |
| 10 | hsa-miR-4705 | 4.39 | Up |
| 11 | hsa-miR-1268b | 3.66 | Up |
| 12 | hsa-miR-187-3p | 3.52 | Up |
| 13 | hsa-miR-4773 | 3.12 | Up |
| 14 | hsa-miR-4458 | 2.84 | Up |
| 15 | hsa-miR-3617-5p | 2.74 | Up |
| 16 | hsa-miR-548d-5p | 2.74 | Up |
| 17 | hsa-miR-1255b-5p | 2.59 | Up |
| 18 | hsa-miR-210-3p | 2.55 | Up |
| 19 | hsa-miR-944 | 2.36 | Up |
| 20 | hsa-miR-504-5p | 2.25 | Up |
| 21 | hsa-miR-210-5p | 2.05 | Up |
| 22 | hsa-miR-4788 | 2.02 | Up |
| 23 | hsa-miR-4521 | 1.94 | Up |
| 24 | hsa-miR-3611 | 1.86 | Up |
| 25 | hsa-miR-1247-3p | 1.79 | Up |
| 26 | hsa-miR-141-5p | 1.61 | Up |
| 27 | hsa-miR-151b | 1.57 | Up |
| 28 | hsa-miR-548h-5p | 1.54 | Up |
| 29 | hsa-miR-193a-3p | 1.52 | Up |
| 30 | hsa-miR-1185-2-3p | 1.31 | Up |
| 31 | hsa-miR-151a-5p | 1.29 | Up |
| 32 | hsa-miR-107 | 1.14 | Up |
| 33 | hsa-miR-3173-5p | 1.08 | Up |
| 34 | hsa-miR-29b-3p | 1.05 | Up |
| 35 | hsa-miR-450a-1-3p | 1.01 | Up |
| 36 | hsa-miR-874-3p | -1.02 | Down |
| 37 | hsa-miR-549a-3p | -1.03 | Down |
| 38 | hsa-miR-216a-5p | -1.07 | Down |
| 39 | hsa-miR-301b-3p | -1.12 | Down |
| 40 | hsa-miR-549a-5p | -1.17 | Down |
| 41 | hsa-miR-378a-3p | -1.18 | Down |
| 42 | hsa-miR-18a-3p | -1.21 | Down |
| 43 | hsa-miR-1228-5p | -1.24 | Down |
| 44 | hsa-miR-500a-5p | -1.25 | Down |
| 45 | hsa-miR-6720-3p | -1.26 | Down |
| 46 | hsa-miR-605-3p | -1.39 | Down |
| 47 | hsa-miR-877-5p | -1.50 | Down |
| 48 | hsa-miR-708-5p | -1.62 | Down |
| 49 | hsa-miR-146b-5p | -1.73 | Down |
| 50 | hsa-miR-495-5p | -1.77 | Down |
| 51 | hsa-miR-26a-1-3p | -1.81 | Down |
| 52 | hsa-miR-4787-3p | -1.94 | Down |
| 53 | hsa-miR-942-3p | -1.94 | Down |
| 54 | hsa-miR-6511a-3p | -1.98 | Down |
| 55 | hsa-miR-708-3p | -2.03 | Down |
| 56 | hsa-miR-320d | -2.17 | Down |
| 57 | hsa-miR-12136 | -2.27 | Down |
| 58 | hsa-miR-4485-3p | -2.39 | Down |
| 59 | hsa-miR-877-3p | -2.39 | Down |
| 60 | hsa-miR-146b-3p | -2.46 | Down |
| 61 | hsa-miR-3679-5p | -2.65 | Down |
| 62 | hsa-miR-6803-3p | -2.88 | Down |
| 63 | hsa-miR-4423-3p | -3.07 | Down |
| 64 | hsa-miR-6511b-5p | -3.07 | Down |
| 65 | hsa-miR-378d | -3.09 | Down |
| 66 | hsa-miR-548am-3p | -3.39 | Down |
| 67 | hsa-miR-548ay-5p | -3.71 | Down |
| 68 | hsa-miR-4728-5p | -7.10 | Down |
| 69 | hsa-miR-512-3p | -7.10 | Down |
| 70 | hsa-miR-592 | -7.10 | Down |
| 71 | hsa-miR-6789-3p | -7.10 | Down |
| 72 | hsa-miR-6833-3p | -7.10 | Down |
| 73 | hsa-miR-6858-3p | -7.10 | Down |
| 74 | hsa-miR-10399-3p | -7.42 | Down |
| 75 | hsa-miR-1237-3p | -7.42 | Down |
| 76 | hsa-miR-3161 | -7.42 | Down |
| 77 | hsa-miR-3174 | -7.42 | Down |
| 78 | hsa-miR-6854-5p | -7.68 | Down |
| 79 | hsa-miR-1257 | -7.91 | Down |
| 80 | hsa-miR-1228-3p | -8.10 | Down |
| 81 | hsa-miR-4709-3p | -8.10 | Down |
| 82 | hsa-miR-1268a | -13.42 | Down |
